# Auxin and nitric oxide control indeterminate nodule formation

**DOI:** 10.1186/1471-2229-7-21

**Published:** 2007-05-08

**Authors:** Youry Pii, Massimo Crimi, Giorgia Cremonese, Angelo Spena, Tiziana Pandolfini

**Affiliations:** 1 Dipartimento Scientifico Tecnologico, University of Verona, Verona, Italy

## Abstract

**Background:**

Rhizobia symbionts elicit root nodule formation in leguminous plants. Nodule development requires local accumulation of auxin. Both plants and rhizobia synthesise auxin. We have addressed the effects of bacterial auxin (IAA) on nodulation by using *Sinorhizobium meliloti *and *Rhizobium leguminosarum *bacteria genetically engineered for increased auxin synthesis.

**Results:**

IAA-overproducing *S. meliloti *increased nodulation in *Medicago *species, whilst the increased auxin synthesis of *R. leguminosarum *had no effect on nodulation in *Phaseolus vulgaris*, a legume bearing determinate nodules. Indeterminate legumes (*Medicago *species) bearing IAA-overproducing nodules showed an enhanced lateral root development, a process known to be regulated by both IAA and nitric oxide (NO). Higher NO levels were detected in indeterminate nodules of *Medicago *plants formed by the IAA-overproducing rhizobia. The specific NO scavenger cPTIO markedly reduced nodulation induced by wild type and IAA-overproducing strains.

**Conclusion:**

The data hereby presented demonstrate that auxin synthesised by rhizobia and nitric oxide positively affect indeterminate nodule formation and, together with the observation of increased expression of an auxin efflux carrier in roots bearing nodules with higher IAA and NO content, support a model of nodule formation that involves auxin transport regulation and NO synthesis.

## Background

The phytohormone auxin (indole-3-acetic acid, IAA) mediates several processes in plant growth and development such as tropic responses to light and gravity, general root and shoot architecture, organ patterning and vascular development [1]. A role for IAA in nodule development was first postulated in 1936 by Thimann [2], supported by the observation that root nodules have a higher IAA content than uninfected root tissue. Studies on nodule development performed with natural (flavonoids) and artificial (e.g. NPA) inhibitors of auxin transport, as well as direct and indirect measurements of IAA, have indicated that auxin accumulates at the site of nodule initiation during nodule formation [3-5].

Free-living rhizobia synthesize IAA [6] and most likely they retain a similar capacity to synthesize IAA during nodulation, because a positive correlation between IAA production in liquid culture and IAA content of the nodules has been demonstrated by using *Bradyrhizobium japonicum *mutants with different IAA synthesising capacities [7]. Several publications have addressed the putative role of auxin produced by rhizobia in determinate nodule development and function [7-9]. 5-methyltryptophan-resistant mutants of *B. japonicum *that overproduce IAA caused, in comparison with wild type rhizobia, a lower nodule mass and a lower number of nodules in soybean [7]. However, another study [9] has shown that inoculation of soybean plants with a tryptophan catabolic mutant of *B. japonicum *that produced elevated amounts of IAA and IPA (indolyl-3-pyruvic acid) increases nodule volume and root weight compared to inoculation with wild type bradyrhizobia. A promoting effect of IAA on determinate nodule formation was also suggested by the observation that IAA-deficient *B. japonicum *mutants produced significantly less nodules than wild type strains [8]. To our knowledge, the effects of increased or reduced IAA synthesis by rhizobia on indeterminate nodule formation has not been investigated by genetic methods.

Nodule organogenesis and lateral root formation display some similarities. Both organs require auxin at development of the primordia and for the differentiation of the vasculature [10,11]. Furthermore lateral root initiation involves the formation of a dynamic auxin gradient in the primordia. Auxin gradient is formed by cellular efflux and requires asymmetrically localized IAA transporters, called PIN proteins [12]. The current model for nodule initiation is also based on the formation of an asymmetric auxin gradient [10].

The auxin signalling pathway and the role of downstream effectors have received great attention in the last years [13]. Recent experimental evidence has shown that NO plays a role in both lateral and adventitious root initiation [14,15]. In auxin-induced adventitious root formation, NO acts as a second messenger and operates downstream of IAA [15].

In this report, we have used *Sinorhizobium meliloti *and *Rhizobium leguminosarum *expressing an auxin-synthesising chimeric operon (*rolAp-iaaMtms2*) to study the effects of rhizobia-derived auxin on nodule formation. We show that auxin synthesised by rhizobia promotes nodulation and host root growth in plants bearing indeterminate nodules whilst no effect was observed in plants bearing determinate nodules. Furthermore, we show that NO is involved in both indeterminate nodule formation and lateral root growth.

## Results

### Expression of *rolAp-iaaMtms2 *in *S. meliloti *increases nodule IAA content and alters root IAA polar transport

**Figure 1:**
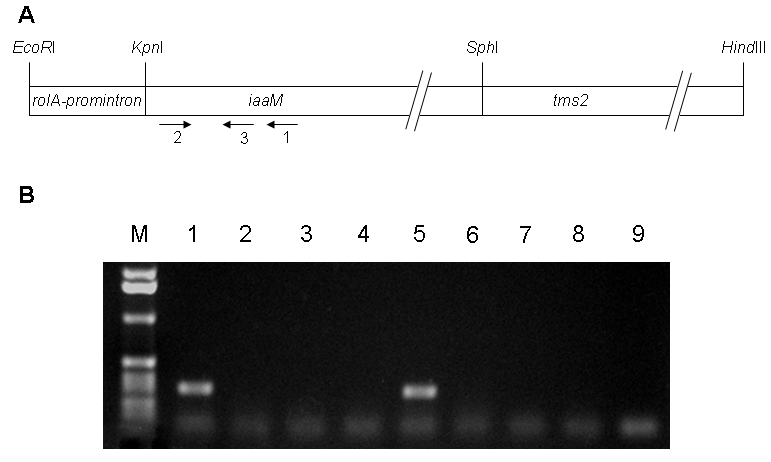
**Expression of *rolAp-iaaMtms2 *construct in indeterminate root nodules**. A. Schematic drawing of the chimeric operon. Restriction endonuclease sites used for chimeric operon construction are reported. B. Agarose gel electrophoresis of RT-PCR product obtained from total RNA extracted from nodules (lanes 1 and 5) formed by *S. meliloti *IAA strain in *M. truncatula *and *M. sativa*, respectively. Lanes 3 and 7, RT-PCR performed on total RNA extracted from nodules induced by the control strain in *M. truncatula *and *M. sativa*, respectively. Lanes 2 and 4, RNA from nodules of *M. truncatula *induced by IAA and control strain, amplified without reverse transcriptase; lanes 6 and 8, RNA from nodules of *M. sativa *induced by IAA and control strain, amplified without reverse transcriptase. Lane 9, no-template control. The position of the primers used in RT-PCR analysis is indicated by arrows in the schematic drawing reported in panel A.

In order to increase the auxin biosynthetic capacity of *S. meliloti*, we engineered a chimeric construct (Fig. 1A) containing the *iaaM *gene from *Pseudomonas syringae *pv. *savastanoi *and the *tms2 *gene from *Agrobacterium tumefaciens *as a bicistronic unit under the control of the prokaryotic promoter (promintron) of the *rolA *gene of *Agrobacterium rhizogenes *[16,17]. The *iaaM *gene codes for a tryptophan monoxygenase, which converts tryptophan to indol-3-acetamide (IAM), while the *tms2 *gene codes for a hydrolase involved in the conversion of IAM to IAA. The 85 bp-long intron of the T-DNA gene *rolA *has a dual function: it behaves as an intron when the *rolA *gene is expressed in plant cells and acts as a prokaryotic promoter in free-living rhizobia and in bacteroids inside nodules [16,17].

The *rolAp-iaaMtms2 *chimeric operon was mobilized into *S. meliloti *strain 1021 to generate an auxin-overproducing strain (hereafter referred to as the IAA strain). RT-PCR analysis, carried out on total RNA extracted from 40 day-old nodules of *Medicago truncatula *and *Medicago sativa *plants infected by the IAA strain, demonstrated that the *rolAp-iaaMtms2 *chimeric operon is transcribed in mature nodules (Fig. 1B).

**Figure 2:**
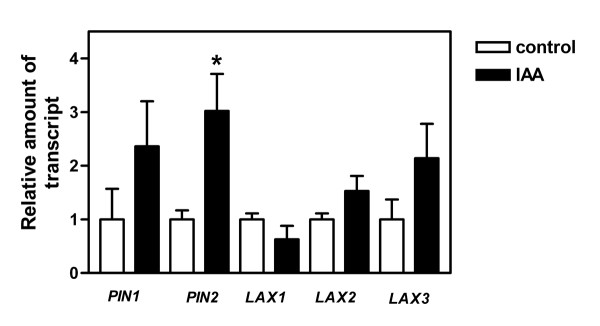
**Expression of auxin carrier genes in *Medicago truncatula *roots**. Expression levels of auxin efflux (PIN1 and PIN2) and influx (LAX1, LAX2 and LAX3) carrier genes were evaluated by quantitative RT-PCR (QRT-PCR). The expression levels were normalized using actin as the endogenous control gene. The QRT-PCR analysis was performed using a ABI Prism 7000 Sequence Detection System. Relative transcript level is the ratio between the expression levels in roots of plant nodulated by the IAA strain and roots of plant nodulated by the control strain. Relative transcript levels were calculated according to manufacturer's recommendations, using the formula 2^-(ΔCtiaa-ΔCtc)^, where ΔCtiaa and ΔCtc is the difference between the threshold cycle of the gene tested and the threshold cycle of actin in IAA and control samples, respectively. The significance of the differences between control and IAA expression levels was evaluated using a Student's *t *test (n = 3). Mean values ± SE are reported. *, P < 0.05. Control: plants nodulated by the control strain. IAA: plants nodulated by the IAA strain.

The total (free and conjugated) IAA concentration of root nodules (1 g FW nodules collected from 40 day-old plants) was measured by GC-MS using deuterated IAA as an internal standard. In control nodules of *M. sativa*, the concentration of IAA was 0.12 nmol/g FW, whereas in extracts obtained from 1 g FW of *M. truncatula *control nodules, IAA was undetectable. Considering the detection limits of the method, we estimate that the value is below 0.010 nmoles for 1 g of tissue. In nodules of *M. sativa *and *M. truncatula *plants infected by the *S. meliloti *IAA strain, the IAA concentration was 1.2 and 1.14 nmol/g FW, respectively. Thus, the expression of the *rolAp-iaaMtms2 *chimeric operon in bacteroids resulted in at least a 10-fold increase in root-nodule auxin content in *Medicago*. The polar transport of auxin, which is crucial for almost all auxin-related developmental processes, is based on the action and the asymmetric distribution of specific auxin influx and efflux carriers [18]. To investigate whether the auxin derived from rhizobia can affect the expression of auxin transporters, we have compared the steady state mRNA levels of selected putative influx and efflux carriers in *M. truncatula *plants bearing IAA overproducing and control nodules. Several members of the *LAX *and *PIN *gene families, directly involved in auxin transport, have been identified in *M. truncatula *[10,19]. In particular, we analysed the expression of three auxin influx carrier genes, *MtLAX1*, *MtLAX2 *and *MtLAX3*, known to be expressed in nodulating roots [10] and two efflux facilitators *MtPIN *genes: *MtPIN2 *expressed only in roots and *MtPIN1*, expressed in both roots and aerial parts [19] (Fig. 2).

The steady state mRNA levels of *MtPIN2 *were significantly higher in roots nodulated by IAA rhizobia compared to roots nodulated by control rhizobia (Fig. 2). The steady state mRNA levels of *MtPIN1 *and the three influx carriers were not significantly modified. *MtPIN2 *expression was not detectable in shoots, as already observed by Schnabel and Frugoli [19]. The mRNA levels of the other auxin transporter genes did not differ in the shoots of plants nodulated with either the IAA or control strains (data not shown).

### IAA synthesised by rhizobia promotes nodulation and root development in legumes with indeterminate nodules

**Figure 3:**
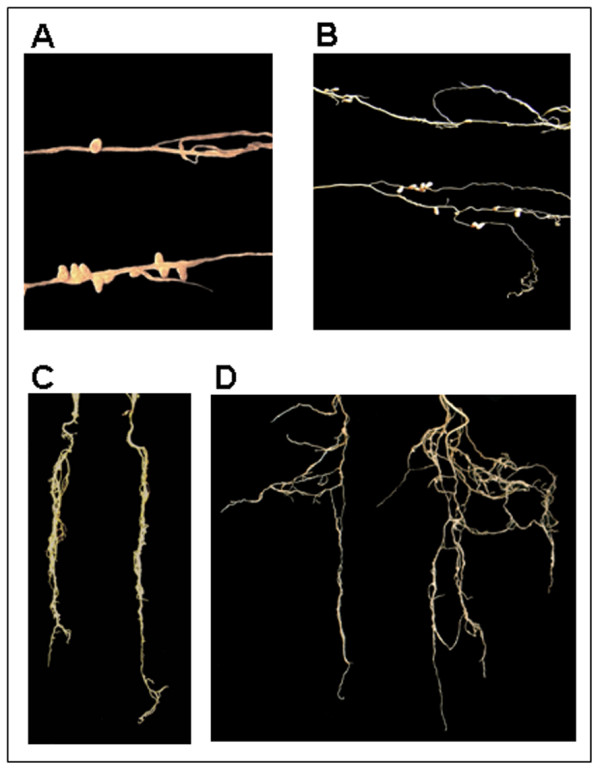
**Phenotypes of *M. truncatula *and *M. sativa *plants nodulated with *S. meliloti *IAA or control strain**. A. *M. truncatula *root nodules: nodules induced by *S. meliloti *IAA strain (bottom) and nodules induced by the *S. meliloti *control strain (top). B. *M. sativa *root nodules: nodules induced by the *S. meliloti *IAA strain (bottom) and nodules induced by *S. meliloti *control strain (top). C. *M. truncatula *roots of plants nodulated by the IAA strain (right) and roots of plants nodulated by the control strain (left) D. *M. sativa *roots of plants nodulated by the IAA strain (right) and roots of plants nodulated by the control strain (left).

**Figure 4:**
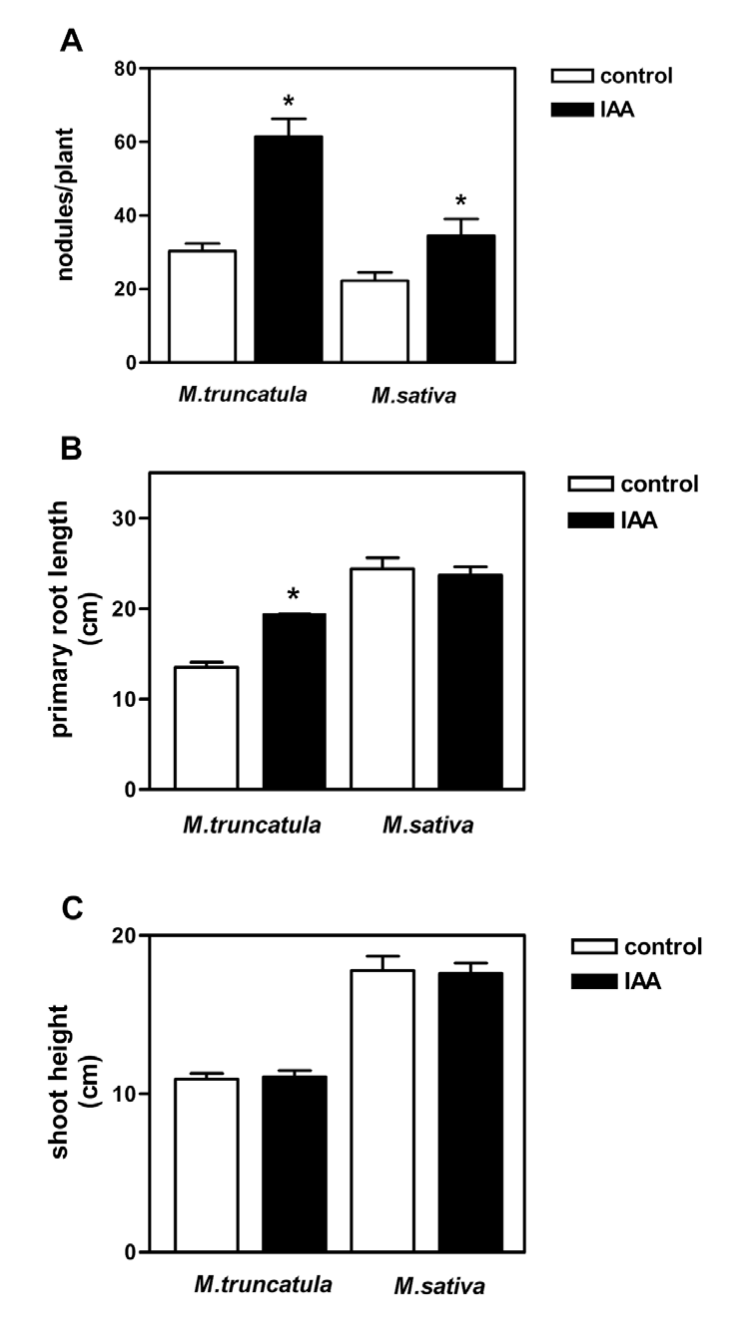
**Effects of increased rhizobial IAA biosynthesis on nodulation and growth of *Medicago *plants**. A. Number of nodules per plant. B. Primary root length. C. shoot height. The values reported are means ± SE (n = ≥22). The experiment was repeated three times with the same results. *, P < 0.05. Control: plants nodulated by the control strain. IAA: plants nodulated by the IAA strain.

Root growth, nodule number and shoot growth of plants inoculated with either IAA or control *S. meliloti *strains were evaluated 40 days after germination. In *M. truncatula*, the average number of nodules per plant was doubled in plants infected by the IAA strain compared to plants infected with the control strain (Fig. 3A and Fig. 4A).

A stimulatory effect on nodulation was also observed in *M. sativa *where the mean number of nodules per plant produced by IAA strain was approximately 50% higher than in plants nodulated by the control strain (Fig. 3B and Fig. 4A). The weight and size of the nodules were on average identical regardless of whether plants were nodulated by the IAA or control strain (Fig. 3A and Fig. 3B and data not shown).

Both *M. truncatula *and *M. sativa *plants bearing IAA-overproducing nodules had a more developed root apparatus in comparison with plants nodulated by the control strain (Fig. 3C and Fig. 3D). The lateral root growth (calculated as weight of total root apparatus/cm of primary root) was on average two times higher in *M. truncatula *plants nodulated by the IAA strain (mean value ± SE = 26 ± 2.6 mg/cm; n = 18) than those nodulated by the control strain (mean value ± SE = 13 ± 1.2 mg/cm, n = 18) (see also additional file 1). A similar increase in lateral root growth was observed in *M. sativa *(Fig. 3D).

We have also investigated the relationship between the number of lateral roots and the number of nodules present on them. The two parameters were significantly correlated in plants inoculated with IAA strain but not in plants inoculated with the control strain (see additional file 2). Altogether, these data suggest that IAA-overproducing rhizobia have a greater capacity to nodulate lateral roots and also a positive effect on lateral root formation.

When *Medicago *plants were grown under conditions that limit root growth – and in particular lateral root growth- (i.e. in 15 ml plastic tubes), a higher density of nodules was observed in plants inoculated by IAA strain compared to those inoculated by control strain (mean values ± SD: 0.12 ± 0.04 and 0.08 ± 0.02 nodules/mg root FW with IAA and control strain, respectively; P < 0.05, n = 12). Thus under conditions that limit root growth, IAA strain still retains a higher capacity to induce nodule formation. This suggests that the increase in lateral root growth is most probably a consequence of the increased synthesis of IAA in the nodules.

The primary root of *M. truncatula *plants bearing IAA-overproducing nodules was on average 40% longer than control plants, whereas in *M. sativa *nodulated by IAA strain, primary root growth was unchanged (Fig. 3C, Fig. 3D and Fig. 4B). No difference in growth of the aerial parts (measured as shoot height) was observed between plants nodulated by either the IAA or control strains. (Fig. 4C). This observation was confirmed by the evaluation of dry matter production and total protein concentration in aerial parts that did not vary in all the experiments (data not shown)

### Nodulation of legumes with determinate nodules is not affected by IAA-overproducing rhizobia

**Figure 5:**
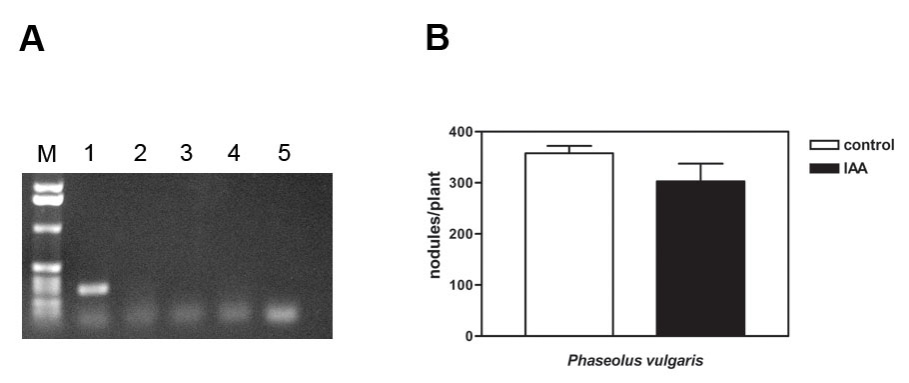
**Expression of the *rolAp-iaaMtms2 *chimeric operon in determinate root nodules**. A. Agarose gel electrophoresis of RT-PCR product obtained from total RNA extracted from bean nodules (lane 1) formed by *Rhizobium leguminosarum *IAA strain. Lane 3, RT-PCR analysis performed on total RNA extracted from bean nodules induced by the control strain. Lanes 2 and 4 reaction without reverse transcriptase performed on total RNA extracted from bean nodules induced by IAA and control strain, respectively. Lane 5, no-template control. B. Number of nodules per plant. The values reported are means ± SE (n = 13). The mean values are not statistically different.

The *rolA *promintron is able to drive bacterial gene expression in determinate nodules (*Phaseolus vulgaris*) as shown by the *rolAp-GUS *gene construct (data not shown). In order to study the effect of increased IAA rhizobial synthesis on determinate nodules, *R. leguminosarum bv. phaseoli *harbouring the *rolAp-iaaMtms2 *construct was used to inoculate *Phaseolus vulgaris *plants. Expression of the *rolAp-iaaMtms2 *chimeric operon in bean nodules was proved by RT-PCR analysis (Fig. 5A).

Expression of the *rolAp-iaaMtms2 *operon in mature determinate nodules of 30 days-old *Phaseolus vulgaris *plants results in a ten times higher concentration of IAA (0.034 nmol/g FW) compared to control nodules (0.003 nmol/g FW). Differently from *Medicago *species where an increase of IAA in the nodules was associated with enhanced nodulation and root growth (Fig. 3 and Fig. 4), the number of nodules and the root growth did not significantly differ in bean plants nodulated by IAA-overproducing rhizobia compared with control strain (Fig. 5B and data not shown).

### NO is involved in the formation of indeterminate nodules

**Figure 6:**
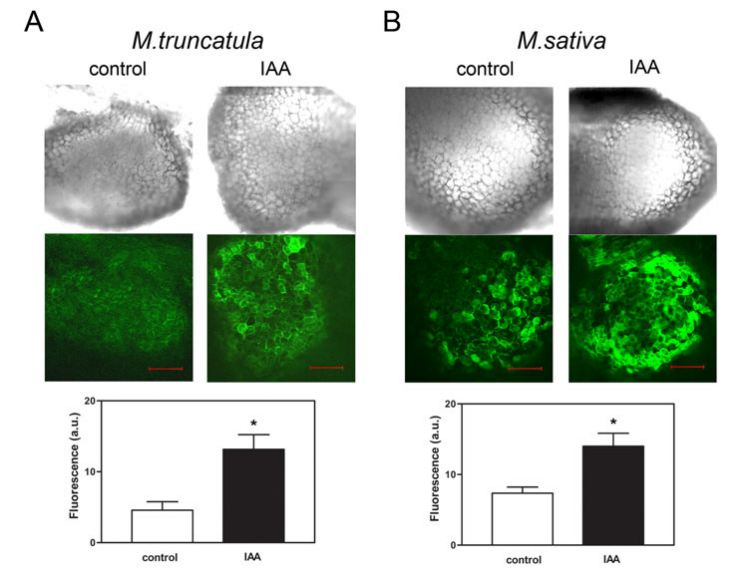
**Evaluation of NO production in nodules**. A. *M. truncatula *nodulated with *S. meliloti *IAA or control strain B. *M. sativa *nodulated with *S. meliloti *IAA or control strain. Upper panels: Microscopy. Bright-field images of the nodules (top) and the confocal laser scanning microscopy (CLSM) detection of endogenous NO in the same nodules (bottom, excitation at 488 nm, emission at 505–530 nm). Bars indicate 200 μm. Photographs are representative of results obtained from the analysis of nodules in three independent experiments. Lower panels: Analysis of fluorescence intensities in nodules induced by IAA and control *S. meliloti *strain. Results are means ± SE (from at least 15 nodules); all data are statistically significant (*P *< 0.05). IAA: nodules produced by the *S. meliloti *IAA strain. Control: nodules produced by the *S. meliloti *control strain.

We also evaluated endogenous NO production in nodules, from *Medicago *plants produced by either the control or the IAA *S. meliloti *strain, loaded with the permeable NO-sensitive dye fluorophore 4,5-diaminofluorescein diacetate (DAF-2-DA). Figure 6 demonstrates that NO production is significantly increased in both *M. truncatula *and *M. sativa *IAA-overproducing nodules. The increase in NO production was about 3 times and 2 times higher in *M. truncatula *and *M. sativa*, respectively (Fig. 6A and Fig. 6B). The NO level in control nodules was higher in *M. sativa *compared to *M. truncatula *(Fig. 6A and Fig. 6B).

Some plant associated bacteria can generate NO from the conversion of L-arginine to L citrulline through an NO synthase activity [20]. Using DAF-2-DA to evaluate NO production in free-living *S. meliloti *at stationary phase of growth, we have observed that IAA-overproducing *S. meliloti *grown under aerobically conditions with ammonium salts as nitrogen source, generate, after arginine addition, a fluorescence signal similar to control strain (our observation). In agreement with this observation, NO synthase-like activity of IAA-overproducing and control rhizobia was not significantly different (see additional file 3).

This indicates that free living *S. meliloti *is able to produce NO and that wild type and IAA strain do not differ in NO production. This observation suggests that bacteroids can contribute to NO production in the nodule.

**Figure 7:**
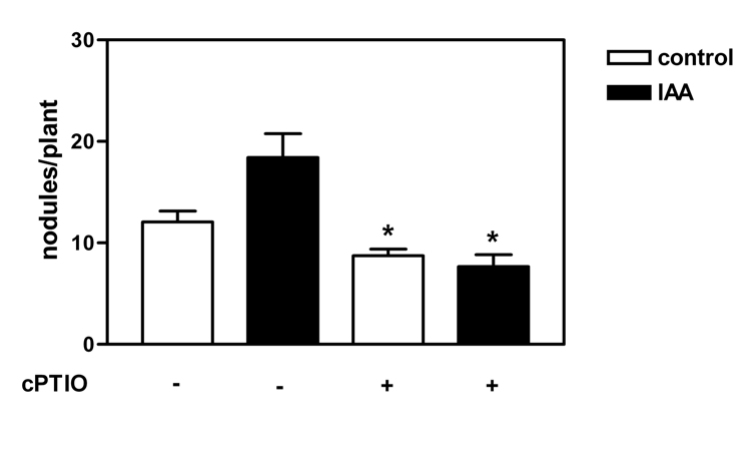
**Effects of the NO scavenger cPTIO on nodulation**. Nodule number of *Medicago truncatula *plants inoculated by IAA and control strain and treated with 1 mM cPTIO. Results are means ± SE (n = at least 12). cPTIO treatments are significantly different from respective controls, (P < 0.05). IAA: nodules produced by the *S. meliloti *IAA strain. Control: nodules produced by the *S. meliloti *control strain.

In order to assess a possible link between NO and indeterminate nodule formation, we tested the effect of the NO scavenger, cPTIO on *M. truncatula *plants inoculated by IAA and control *S. meliloti *strains. The plants, grown in plastic tubes on perlite supplemented with N-free nutrient solution, were treated with 1 mM cPTIO 2, 24 and 48 h after rhizobia inoculation. NO depletion by treatment with cPTIO caused a significant reduction in nodule number (Fig. 7) in plants inoculated with either IAA-overproducing or control rhizobia. This finding demonstrates that NO depletion inhibited indeterminate nodule formation and completely abolished the auxin stimulatory effect on nodulation. Nitric oxide depletion inhibited the increase in lateral root growth caused by IAA-overproducing strain (data not shown), confirming previous data on the role played by NO in lateral root formation in tomato [14]. Primary root length and shoot growth were not affected by 1 mM cPTIO. Furthermore, the treatment with cPTIO has no effect on *S. meliloti *growth (see additional file 4).

## Discussion

The formation of N_2_-fixing root nodules in leguminous plants requires a complex exchange of signals between the host and the compatible rhizobia strain. Phytohormones, and in particular auxin, have been implicated in this process. Our data demonstrate that rhizobium-derived auxin promotes indeterminate nodule formation (*Medicago *sp.), whilst an increased synthesis of IAA within rhizobia does not affect determinate nodule formation (*Phaseolus vulgaris*).

The effects of IAA-overproducing and IAA-deficient rhizobia mutants on nodulation has been investigated in several previous studies that have reported rather contrasting results about the role of rhizobial IAA synthesis in nodule development [7-9]. Moreover, the results presented by those studies are somehow difficult to interpret due to the limited molecular characterization of the mutants. Our experimental approach is novel in that we have used rhizobia genetically engineered for a new (*iaaMtms2*) auxin biosynthetic pathway to address the role of rhizobia-derived auxin. Thus, the IAA-overproducing rhizobia strain differs from the control strain only for the increased auxin synthesising capacity. The *Sinorhizobium meliloti *strain that overproduces auxin has an enhanced ability to nodulate both *M. sativa *and *M. truncatula*, which results in a 50% and 100%, respectively, increase in the number of nodules per plant compared to the control strain. The use of the *rolA *promoter [17] to drive *iaaMtms2 *expression enables the synthesis of auxin in bacteroids leading to an increase of auxin content within the nodules. The *rolA *promoter is active also in free living rhizobia [17]. Thus, it is likely that the synthesis of auxin takes place also during early phases of infection (e.g. root-hair curling and formation of the infection thread) and nodule initiation.

In the current model of both determinate and indeterminate nodule organogenesis, the local accumulation of auxin at the site of nodule initiation is thought to stimulate cellular division in the cortex and pericycle [10,4]. The higher auxin synthesising capacity of the IAA strain may facilitate nodule formation by increasing auxin levels within the nodule primordium. This interpretation is somehow in agreement with the observation that a *M. truncatula *supernodulating plant mutant contains three times more auxin than wild type at the site of nodule initiation [21]. Furthermore, the observation that the average size of IAA overproducing and control indeterminate nodules is similar indicates that rhizobia-derived auxin affects mainly nodule formation rather than nodule growth.

The auxin loading model of van Noorden and colleagues [21] suggests that the inhibition of auxin loading from shoot to root is the basis of the autoregulation of nodulation in indeterminate legumes. This mechanism is altered in the *sunn *hypernodulating mutant and consequently auxin continues to be transported from the shoot to the root and sustains supernodulation [21]. In our experimental system, the increased number of nodules obtained in *Medicago *plants is most likely the consequence of extra auxin loaded in the root from the bacteroids within the nodules.

Determinate nodules apparently do not inhibit the auxin transport from the shoot [5] and an hypernodulating plant mutant (soybean *nts*) does not show an increased auxin level in the root [22]. This finding has prompted van Noorden and colleagues [21] to propose that determinate and indeterminate nodules differ in the requirement, transport capacity or regulation of auxin transport [5,21]. In accordance with this hypothesis, our results show that an increased auxin synthesis in bacteroids does not affect determinate nodule formation in *Phaseolus vulgaris*.

*M. truncatula *and *M. sativa *plants bearing IAA overproducing nodules, compared to plants with control nodules, have a more developed root apparatus with abundant lateral roots, a characteristic trait observed in some mutants that overproduce auxin [1]. A striking similarity between lateral root and indeterminate nodule development has been already indicated [4,10]. Moreover, based on the observation of a strong correlation between nodule and lateral root number in pea, a possible overlap during early developmental pathways of the two organs has been suggested by Ferguson et al. [23]. Our data are somehow consistent with this hypothesis [23] since both more nodules and more developed lateral roots are observed in *Medicago *plants nodulated by IAA-overproducing rhizobia. However, in plants inoculated with the control strain we did not find any correlation between the number of lateral roots and the number of nodules present on the lateral roots (see additional file 2). On the other hand, the significant correlation between nodule and lateral root numbers detected in *M. truncatula *plants inoculated with IAA strain (see additional file 2) suggests that IAA-overproducing rhizobia have a greater capacity to nodulate lateral roots besides a positive effect on lateral root formation.

We did not observe any effects of IAA overproducing rhizobia on the growth and biomass production of the aerial parts of *M. truncatula *and *M. sativa*. Thus, under the growth conditions used in the experiments, there is no indication of an increased nitrogen fixation. However, we can not rule out that plants grown under limiting growth conditions might eventually take advantage (i.e. a more efficient water and nutrient uptake) from the more developed root apparatus induced by IAA overproducing rhizobia.

Auxin transport is mediated by asymmetrically-localized auxin influx/efflux facilitators that regulate auxin distribution during root and shoot growth [12,18]. Changes in the expression levels of auxin carriers can affect root development. In this study, we show that plants with IAA-overproducing nodules have increased expression of the root-specific *MtPIN2 *gene, an ortholog of the auxin efflux carrier *PIN2 *of *A. thaliana *[19] that mediates the transport of auxin towards the root elongation zone [18]. An increased *PIN2 *expression has also been reported in the hypernodulating *sickle *mutant of *M. truncatula *[24] and nodulation was shown to be inhibited in *PIN2 *silenced plants [25]. The aforementioned result suggests that the observed changes in nodulation and root growth are most likely the consequence of both the increased concentration of auxin in the nodules and the auxin redistribution in the root tissue.

The involvement of NO in auxin-induced adventitious root formation in cucumber and in lateral root formation in tomato has been recently reported, providing evidence that NO is a component of the auxin signalling pathway in these processes [14,26]. This work shows that NO is produced in root nodules of *M. truncatula *and *M. sativa *and that NO is increased in IAA-overproducing nodules. In plant tissues NO can be generated by enzymatic and non enzymatic systems [27], while rhizobia under anaerobic condition produce NO via the denitrification pathway [28]. According to a recent study [29] enzymes of the denitrification pathway do not contribute to NO generation during nodule development. We have observed that aerobically-grown stationary phase IAA-overproducing and wild type *S. meliloti *produce NO and possess NO synthase-like activity. Thus, NO level in nodules could be the result of both plant and bacterial production. However, no difference in NO production was observed in free living wt and IAA strains. Consequently, the increased NO level present in IAA-overproducing nodules is likely the result of plant NO synthesis induced locally by bacterial IAA. Based on these results, a role for IAA and NO in indeterminate nodule formation is hereby proposed. To our knowledge, the data showing that nodule NO biosynthesis is increased in plants with higher nodulation and that a NO scavenger reduces nodule formation represents the first experimental evidence of NO involvement in the auxin-signalling pathway controlling indeterminate nodule formation.

## Conclusion

The data presented demonstrate, by using rhizobia engineered for a high production of auxin, that an increased bacterial auxin synthesis promotes the formation of indeterminate nodules, whereas it has no effect on determinate nodule formation. We also show that nitric oxide acts as a signal molecule in controlling, either directly and/or indirectly, nodule number in indeterminate legumes. These data indicate that indeterminate nodule formation involves regulation of both auxin and NO signalling.

## Methods

### Bacterial strains

*Sinorhizobium meliloti *1021 is a streptomycin-resistant derivative of wild-type field isolate SU47 [30]. *S. meliloti *was grown at 28°C in LBMC medium (10 g/l tryptone, 5 g/l yeast extract, 10 g/l NaCl, 2.6 mM MgSO_4_, 2.6 mM CaCl_2_) supplemented with streptomycin 200 μg/ml. *Rhizobium leguminosarum bv. phaseoli *was grown at 28°C in YEM medium (mannitol 10 g/l, NaCl 0.1 g/l, MgSO_4 _0.2 g/l, KH_2_PO_4 _0.5 g/l, yeast extract 0.4 g/l).

### Plasmids and gene constructs

Standard techniques were used for the construction of recombinant DNA plasmids. The *rolAp*-*iaaMtms2 *chimeric operon contains the bicistronic unit *iaaMtms2 *under the control of *promintron*, the 85 bp-long intron of *rolA *gene of *Agrobacterium rhizogenes *which has promoter function in bacteria [17]. A 1773 bp-long DNA sequence spanning the coding region (1671 bp) of the *iaaM *gene (GenBank accession n. M11035) from *Pseudomonas syringae pv. savastanoi *[31] and a 1452 bp-long DNA sequence spanning the coding region (1404 bp) of *tms2 *(GenBank accession n. AH003431) from *Agrobacterium tumefaciens *[32], were cloned downstream of the *promintron *sequence (*rolAp*), connected by a 17 bp-long linker sequence. The *rolAp*-*iaaMtms2 *construct was subcloned in the broad-host range plasmid pMB393 [33] and introduced by electroporation into *S. meliloti *1021 and *R.l. phaseoli *to obtain the IAA strains. *S. meliloti *1021 and *R.l. phaseoli *harbouring the pMB393 plasmid containing the *promintron *sequence was used as control strain.

### Plant growth and inoculation

*Medicago truncatula *cv. *Jemalong *and *Medicago sativa *ecotype *Romagnolo *seeds were scarified using fine grade sand paper sheets and sterilized in 5% commercial bleach for 3 min. Seeds were rinsed three times with sterile water and stored on 0.8% agar plates at 4°C for 2 days before placing in a growth chamber at 25°C for 7 days to allow germination. *Phaseolus vulgaris *seeds were sterilized in 12% commercial bleach for 7 minutes, rinsed and then imbibed in sterile water for 1 hour.

Germinated seedlings of *M. truncatula *were transferred in small pots and grown on a sand and perlite mixture (1:1) in a growth chamber at 22°C and 10-h light/14-h dark regimen under fluorescent lights giving an average irradiance of 120 μmol m^-2 ^sec^-1 ^of photosynthetically active radiation (PAR); the relative humidity was 65%. *Medicago sativa *and *Phaseolus vulgaris *seedlings were grown in small pots on sand and perlite mixture (1:1) in a greenhouse at day and night temperatures of 24°C and 18°C, respectively, and 10 h light/14 h dark regimen. Once a week the *Medicago sp*. and *P. vulgaris *seedlings were supplemented with a nitrogen-free nutrient solution (0.13 mM KH_2_PO_4_; 0.3 mM CaCl_2_·2H_2_0; 0.06 mM MgSO_4_·7H_2_O; 0.2 mM K_2_SO_4_; 0.014 mM FeNa EDTA; 1.56 mM H_3_BO_3_; 1.24 mM MnSO_4_·H_2_O; 4.5 mM KCl; 0.11 mM ZnSO_4_·7H_2_O; 0.1 mM CuSO_4_·5H_2_O; 0.32 mM H_2_SO_4_; 2.1 mM Na_2_MoO_4_·2H_2_O). The seedlings were watered with sterile deionized water as necessary.

For plant inoculation, bacteria were grown in liquid medium, collected by centrifugation, washed in sterile water, and then diluted in sterile water to 0.1 OD_600 _(approximately 10^8 ^cfu/ml). Ten ml of this suspension were used to inoculate seedlings at 10 and 24 days after germination. Leaves, roots and root nodules were collected 40 days after germination. At the end of each experiment, the presence of the recombinant plasmids in bacteroids was checked by PCR analysis on total DNA extracted from root nodules.

For cPTIO treatments, *M truncatula *seedlings were transferred after germination in 15 ml test tubes, containing nitrogen-free nutrient solution and perlite (1:2 vol/vol) and grown in a growth chamber at 24°C 16-h light/8-h dark regimen under fluorescent lights giving an average irradiance of 120 μmol m^-2 ^sec^-1 ^PAR; the relative humidity was 65%. Ten days old plants were inoculated using 1 ml of a bacterial suspension to an OD_600 _of 0.1. Two hours after inoculation, 1 ml of 1 mM 2-(4-carboxyphenil)-4,4,5,5,-tetramethylimidazoline-1-oxyl-3oxide (cPTIO) (Sigma, St. Luois, MO, USA) was added to the nutrient solution; 1 ml of distilled water was used for negative controls. This treatment was repeated 24 and 48 hours after inoculation. Nodules were counted 28 days after inoculation. cPTIO (1 mM) has no effect on *S. meliloti *growth (see additional file 4).

### IAA analysis

Root nodules (1 and 5 g FW for *Medicago *and bean plants, respectively) were collected from 40 day-old plants. IAA extraction was carried out as previously described [34]. 100 nmols of D^5- ^IAA were added to the samples, as internal standard.

TMS GC-MS analysis was performed on a Hewlett Packard 5890 instrument equipped with a HP-5 (Agilent technologies) fused silica capillary column (30 m, 0.25 mm ID, helium as carrier gas), with the temperature program: 70°C for 1 min, 70°C→150°C at 20°C/min, 150°C→200°C at 10°C/min, 200°C→280°C at 30°C/min, 280°C for 15 min. The injection temperature was 280°C. Electron Ionisation (EI) mass spectra were recorded by continuous quadrupole scanning at 70eV ionisation energy.

### NO detection

Endogenous NO was detected with the fluorophore 4,5-diamino-fluorescein diacetate (DAF-2-DA). 4-aminofluorescein diacetate (4-AF DA) was used as a negative control, assuming that the green fluorescence detected corresponds to endogenous NO and not to unspecific reactions of the probe. Nodules obtained with the control and IAA strains were incubated with 7.5 μM DAF-2DA (Calbiochem) or with the negative probe 4 AF-DA (Calbiochem) in 20 mM HEPES-NaOH, pH 7.5 (buffer A) for 30 min in the dark at 25°C. Thereafter, nodules were washed three times for 15 min each with buffer A and fluorescence was detected with a Zeiss LSM 510 laser scanning confocal microscope exciting at either 488 or 543 nm. For emission in the green light, fluorescence was examined between 505 and 530 nm, while in the red light, fluorescence was collected at wavelengths >560 nm.

Green fluorescence was quantified by measuring the medium pixel intensity in the confocal images for every single nodule analyzed. All the quantitative data were subjected to statistical evaluation (Student's *t *test). A *P *< 0.05 was considered statistically significant.

### RT-PCR analysis

Total RNA (2 μg) extracted from nodules was treated with 2 units of RQ1 DNase (Promega, Madison, WI) and then used as a template for a reverse transcriptase (Superscript II, Invitrogen, Carlsbad, CA) reaction primed with the oligonucleotide 5'-CTCCGTGTCCACCACACC-3' (Primer 1) complementary to the iaaM coding region +372 and +389 bp. The complementary DNA was amplified with the forward primer 5'-ATGTATGACCATTTTAATTCACCCAGT-3' (Primer 2), corresponding to the region +1/+27 of the iaaM gene (+1 is the initiation of translation), and with the primer 5'-CTGGGAGGAAAGCGCATCGCAC-3' (Primer 3), complementary to the region +283/+304 of the *iaaM *gene.

For quantitative RT-PCR analysis (QRT-PCR), leaf and root samples were frozen in liquid nitrogen immediately after collection and stored at -80°C. Root samples do not include nodules that were detached from the roots before freezing.100 mg of pooled tissues, derived from three different plants, were ground in liquid nitrogen and total RNA was isolated by using Rneasy Plant Mini Kit (QIAGEN), according to the manufacturer's protocol. Five μg of total RNA were treated with 5 units of RQ1 DNase (1 U/μl) (Promega, Madison, WI). All RNA samples were checked for DNA contamination before cDNA synthesis. Comparative PCR analysis was carried out using first strand cDNA obtained with oligo-dT primer and Superscript II (Invitrogen, Carlsbad, CA). The cDNA clones were amplified with gene-specific primers designed to give amplification products ranging from 100 to 150 bp. The nucleotide sequence of the gene-specific primers are the following: *MtPIN1 *forward primer 5'-ATGGCTCTGCTGCTGCTGCTAA-3', reverse primer 5'-TCCAGATTGATCAGACGCTCC-3'; *MtPIN2 *forward primer 5'-GCATGGGCGGTGGAAGTGGTAA-3', reverse primer 5'-TGGAAGGATCAACAGTGCCA-3'; *MtLAX*1 forward primer 5'-AAACAAGGCGAAGAAACAA-3', reverse primer 5'-ACAGCTAAACCAAGCATCAT-3', *MtLAX2 *forward primer 5'-ATGTTGCCACAAAAACAAGG-3', reverse primer 5'-TGAATGAATGATCTTCCACC-3'; *MtLAX3 *forward primer 5'-ATGACTTCTGAGAAAGTTGA-3', reverse primer 5'-CTTAGATAATTTGCCAGTAG-3'; actin forward primer 5'-AGATGCTGAGGATATTCAAC-3', reverse primer 5'-GTATGACGAGGTCGGCCAAC-3'.

The reaction mixture contained Platinum SYBR Green QPCR Supermix-UDG, ROX reference dye to correct for fluorescent fluctuations (Invitrogen, Carlsbad, CA) and 0.4 μM of each primer. UDG and dUTP were included in the mixture to prevent re-amplification of carryover PCR products between reactions. The QRT-PCR was performed with ABI Prism 7000 Sequence Detection System (Applied Biosystems, Foster City, CA) with the following cycling conditions: 2 min at 50°C, 2 min at 95°C, 40 cycles of 95°C for 30 sec, 56°C for 30 sec, 72°C for 30 sec and finally 72°C for 3 min. All quantifications were normalized to the actin gene as an endogenous control. For each amplification reaction, analysis of the product dissociation curve was performed to exclude the presence of non-specific amplification. For each determination of mRNA levels, three cDNA samples derived from three independent RNA extractions were analysed. Relative quantification of transcript levels was carried out as previously described [35].

### Statistical analysis

The mean values ± SE are reported in the figures. Statistical analyses were conducted using a Student's t-test.

## Authors' contributions

YP carried out nodulation experiments and molecular biology analysis; MC performed NO detection in nodules and participated in the manuscript preparation; GC performed the experiments on *Phaseolus*; AS design the gene constructs, participated in manuscript preparation; TP coordinated the study and wrote the manuscript. All authors read and approved the final manuscript.

## Supplementary Material

Additional file 1 **Root apparatus of *M. truncatula***. Picture representing the stretched root apparatus. Click here for file

Additional file 2 **Nodule and lateral root numbers**. Correlation between nodule and lateral root numbers in plants inoculated by IAA and control strain. Click here for file

Additional file 3 **NO synthase-like activity **Measurement of NO synthase-like activity in IAA-overproducing and control *S. meliloti *strains. Click here for file

Additional file 4 **Effect of cPTIO on *S. meliloti *growth**. Effect of cPTIO on *S. meliloti *growth and survival. Click here for file
