# Supplementary material for: Auxin and nitric oxide control indeterminate nodule formation
Source: BMC Plant Biol. 2007 May 8;7:21. doi: 10.1186/1471-2229-7-21 (PMC1878477; doi:10.1186/1471-2229-7-21)
Supplement: Additional file 1 — Root apparatus of M. truncatula. Picture representing the stretched root apparatus. [file 1471-2229-7-21-S1.pdf]

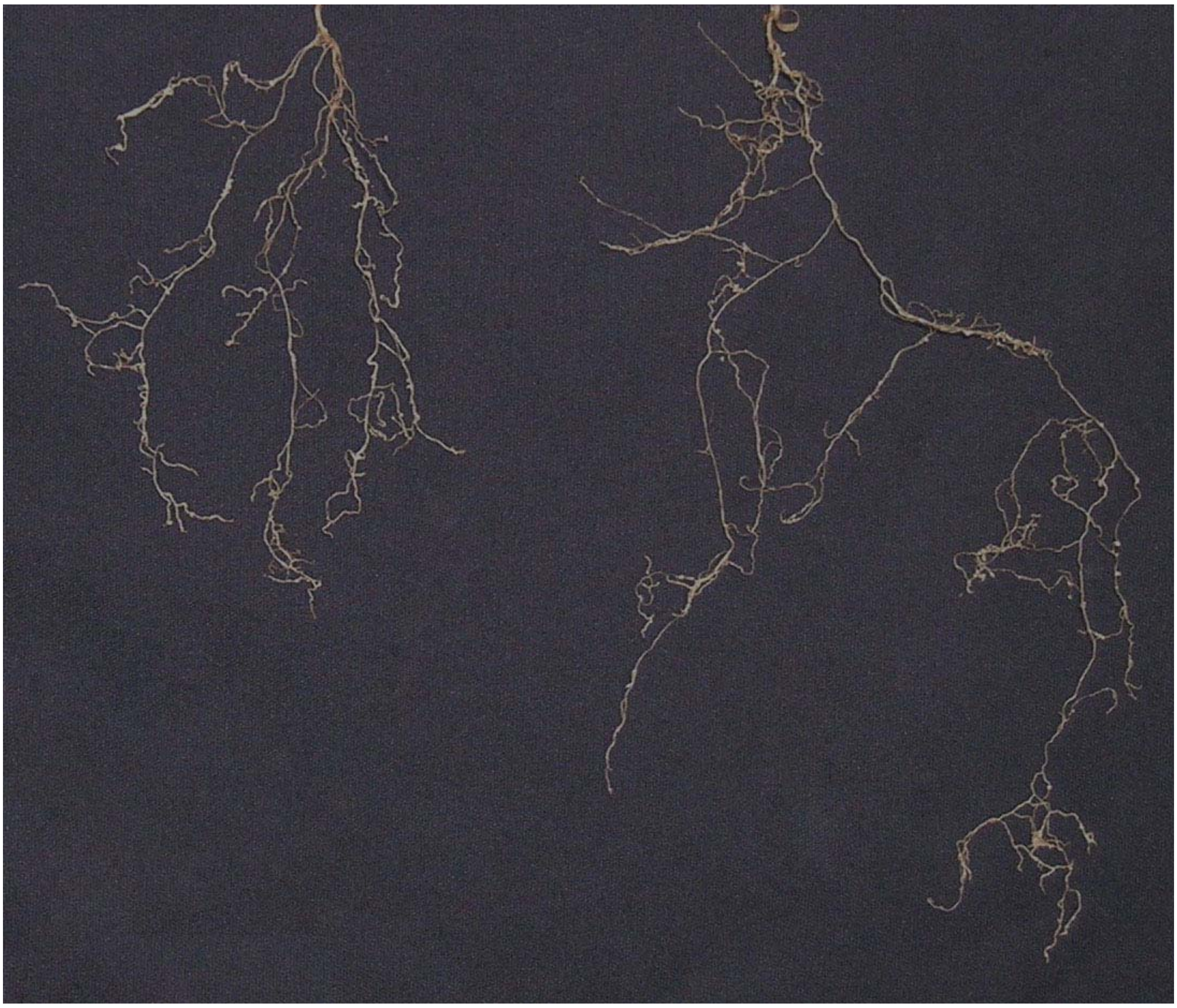

M. truncatula roots of plants nodulated by the control strain (left) and roots of plants nodulated by the IAA strain (right).
