# Supplementary material for: Auxin and nitric oxide control indeterminate nodule formation
Source: BMC Plant Biol. 2007 May 8;7:21. doi: 10.1186/1471-2229-7-21 (PMC1878477; doi:10.1186/1471-2229-7-21)
Supplement: Additional file 2 — Nodule and lateral root numbers. Correlation between nodule and lateral root numbers in plants inoculated by IAA and control strain. [file 1471-2229-7-21-S2.pdf]

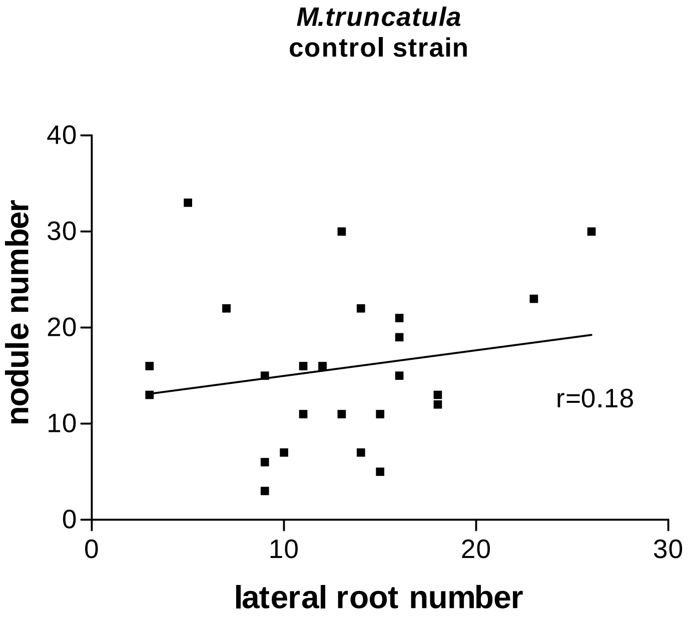

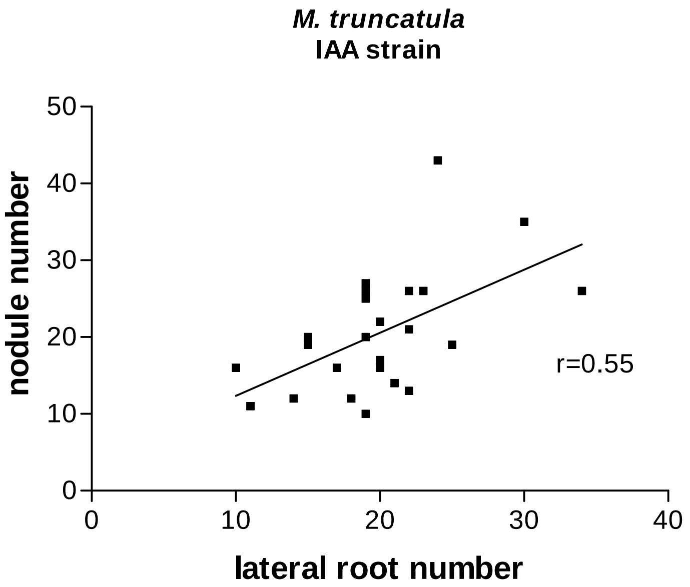

Correlation between the number of nodules present on lateral roots and the number of lateral roots developed by *Medicago truncatula* plants inoculated with IAA and control strain. Nodule and lateral root numbers are significantly correlated ($P<0.01$) only in plants inoculated by IAA strain.
