# Supplementary material for: Auxin and nitric oxide control indeterminate nodule formation
Source: BMC Plant Biol. 2007 May 8;7:21. doi: 10.1186/1471-2229-7-21 (PMC1878477; doi:10.1186/1471-2229-7-21)
Supplement: Additional file 3 — NO synthase-like activity Measurement of NO synthase-like activity in IAA-overproducing and control S. meliloti strains. [file 1471-2229-7-21-S3.pdf]

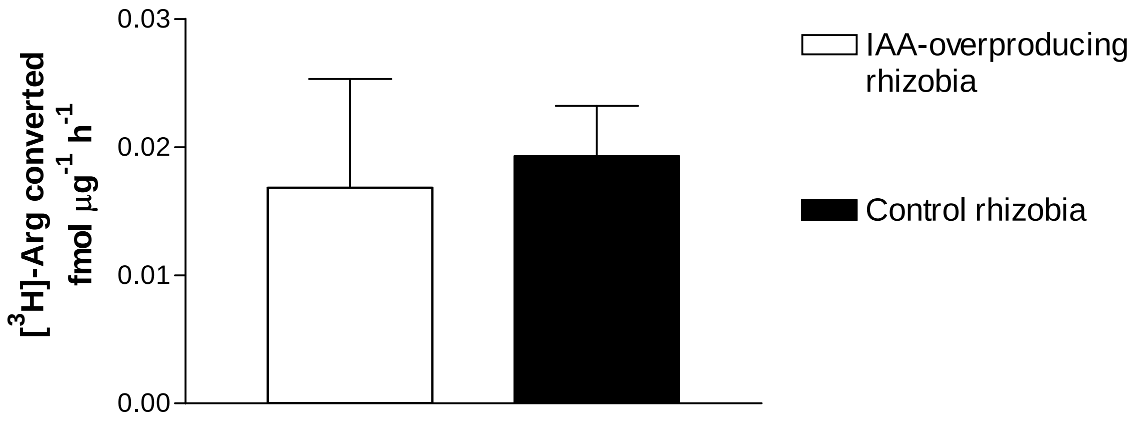

NOS-like activity measured in *S.meliloti* IAA-overproducing and control strains.

NOS-like activity assay

S. meliloti cells were grown on minimal medium (10mM phosphate buffer pH 6.3, 7.4 mM sodium succinate, 2.7 mM D-glucose, 0.8 μM nicotinic acid, 1 mM $MgSO_4$, 0.5 mM $CaCl_2$) with 0.1% NH_4Cl added as nitrogen source. Cultures were incubated with shaking at 180 rpm at 28°C until reaching stationary phase, harvested by centrifugation at 5000 xg for 20 min and resuspended in Homogenization Buffer (25 mM Tris-HCl pH 7.4, 1 mM EDTA, 1 mM EGTA). Cells were disrupted by sonication for 1 min and the soluble fraction was obtained by centrifugation at 13000 xg for 30 min.

NOS-like activity was determined by the method based on the conversion of L-[^{3}H]arginine to L-[^{3}H]citrulline, by using the NOS Activity Assay Kit (Cayman Chemical, Ann Arbor, MI). Total protein concentration was measured by Bradford method and the enzyme activity was expressed as fmol arginine μg protein^{-1}h^{-1}.
