# Supplementary material for: Auxin and nitric oxide control indeterminate nodule formation
Source: BMC Plant Biol. 2007 May 8;7:21. doi: 10.1186/1471-2229-7-21 (PMC1878477; doi:10.1186/1471-2229-7-21)
Supplement: Additional file 4 — Effect of cPTIO on S. meliloti growth. Effect of cPTIO on S. meliloti growth and survival. [file 1471-2229-7-21-S4.pdf]

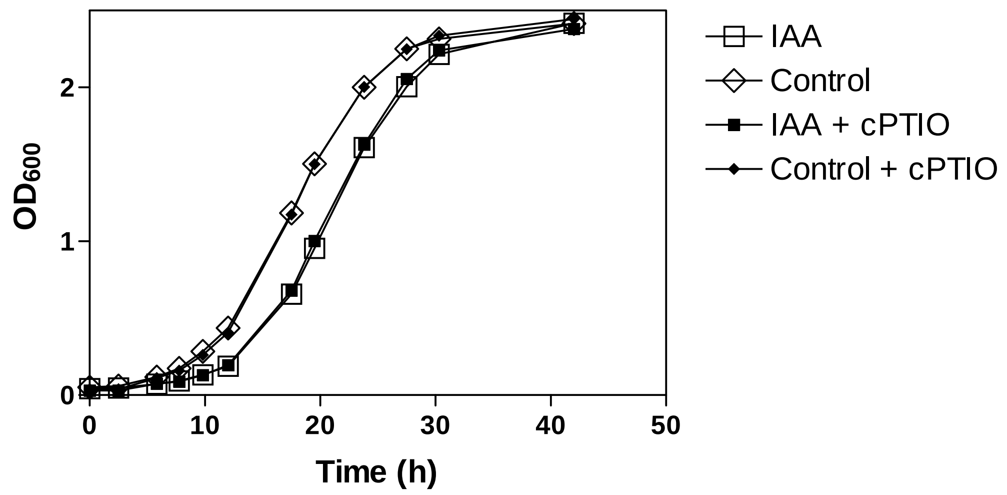

Effect of 1mM *cPTIO* on *S. meliloti* IAA and control strain growth. Bacteria were grown in LB medium supplemented with 2.6 mM $MgSO_4$ and 2.6 mM $CaCl_2$.

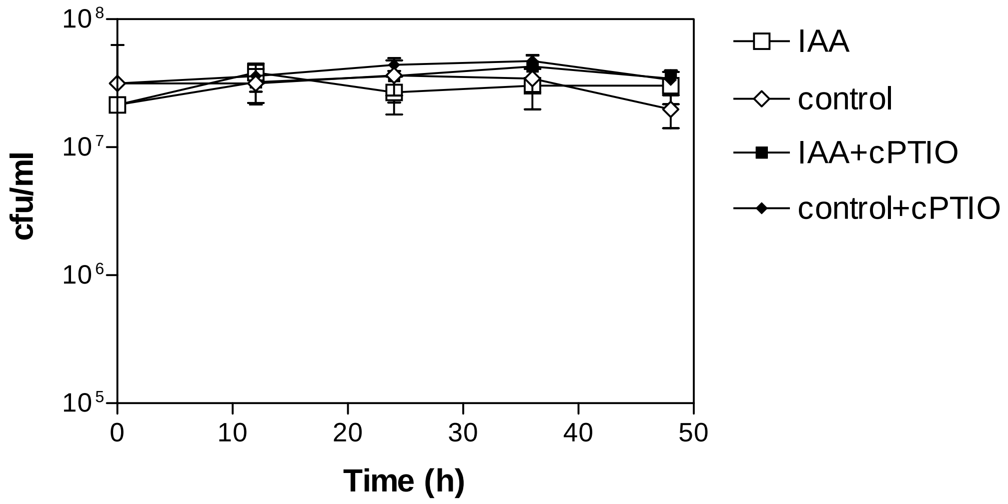

Effect of 1mM *cPTIO* on *S. meliloti* IAA and control strains survival. Bacteria were maintained in minimal medium (M9 salts plus glucose 20%) at 28°C for 48 hours.

A

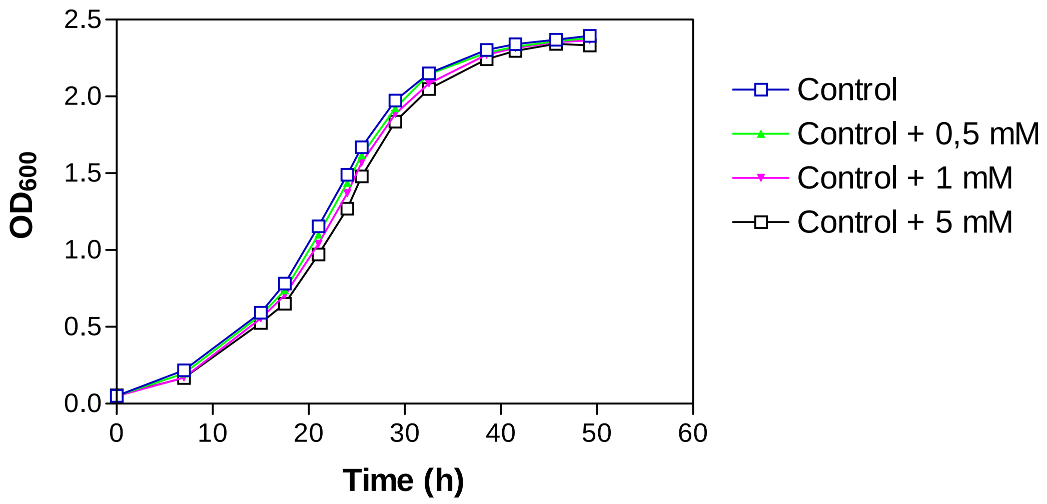

B

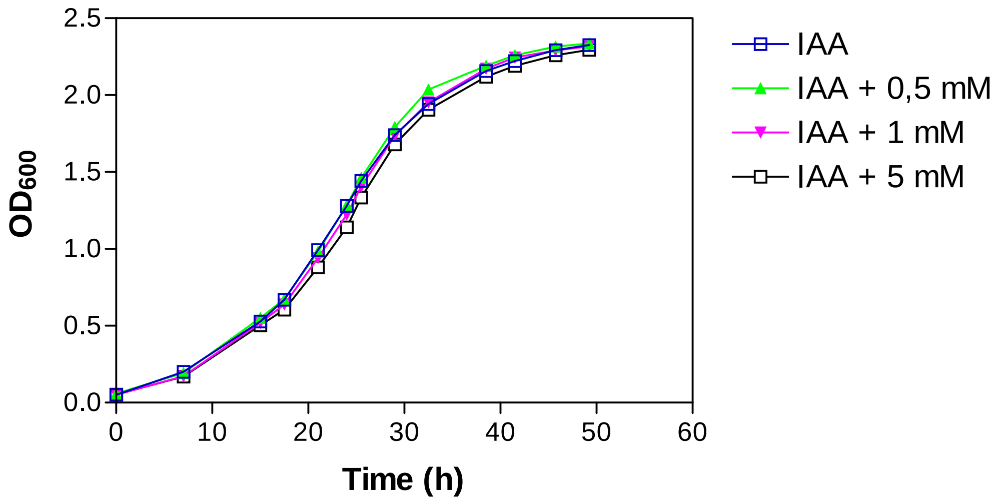

Effect of 0.5, 1 and 5 mM cPTIO on *S. meliloti* control (A) and IAA (B) strains growth . Bacteria were grown in RDM minimal medium (Vincent JM 1970 A manual for the practical study of root nodule bacteria. International Biological Programme, Handbook 15, Blackwell Scientific Publication, London).
